# Editorial: Advances of innovative therapeutic strategies in age-related diseases

**DOI:** 10.3389/fbioe.2023.1288352

**Published:** 2023-09-27

**Authors:** Ling Luo, Bin Wu, Zhiwei Jie, Yiqiang Hu

**Affiliations:** ^1^ Clinical Pharmacology Institute, Pharmaceutical School, Nanchang University, Nanchang, China; ^2^ State Key Laboratory of Materials Processing and Die & Mould Technology, School of Materials Science and Engineering, Huazhong University of Science and Technology, Wuhan, China; ^3^ Department of Orthopaedic Surgery, Sir Run Run Shaw Hospital, Zhejiang University School of Medicine, Hangzhou, China; ^4^ Department of Orthopaedics, Union Hospital, Tongji Medical College, Huazhong University of Science and Technology, Wuhan, China

**Keywords:** age-related diseases, bioinformatics, miRNA, lncRNA, CirRNA, exosomes

The unprecedented increase in life expectancy over the past few decades has led to a rise in the global elderly population. Age-related diseases have also become an increasingly significant social, economic, and medical burden all over the world (GBD 2019 Ageing Collaborators, 2022). This has prompted significant efforts within the scientific and medical research communities to develop and enhance therapies aimed at treating age-related diseases (Esteves et al., 2023). The majority of age-related diseases, including diabetes, Alzheimer’s disease, osteoporosis, as well as cancer, are closely linked to cellular senescence (Abokyi et al., 2023). Cells demonstrate significant phenotypic changes during the aging process, which are driven by alterations in metabolism, chromatin organization, and transcriptional activity. An evident characteristic of aging is the secretion of inflammatory mediators, including various cytokines, chemokines, extracellular matrix proteins, and growth factors, collectively referred to as the senescence-associated secretory phenotype (Weng et al., 2022; Spinelli et al., 2023). In order to extend a healthy lifespan and mitigate the burden of age-related diseases among the elderly population, researchers are exploring ways to slow down the progression of these diseases and to develop effective treatment approaches. This Research Topic focuses on the development of innovative therapeutic strategies in age-related diseases and is expected to bring new perspectives to age-related disease treatment.

Osteosarcoma is the most common primary malignant bone tumor and is characterized by a high degree of local invasiveness and a tendency to metastasize (Chen et al., 2021). The long-term survival rate is poor in patients with osteosarcoma. Therefore, understanding its pathogenesis and developing new treatment methods are still the main unmet needs (Yang et al., 2021). MicroRNA (miRNA) are highly conserved and stable small non-coding RNA molecules that are generally believed to be potential biomarkers and potential therapeutic targets for tumor, including osteosarcoma (Lei et al., 2020; Nail et al., 2023). Han et al. used the bioinformatics methods to find that miR-449a is downregulated in osteosarcoma and plays an important role in the mechanism of osteosarcoma by targeting CCNB1. They found that the overexpression of miR-449a could inhibit SaOS-2 cell proliferation. The research revealed that inhibition of miR-449a in osteosarcoma cells promotes cell proliferation and inhibits its apoptosis. Their results illustrated that CCNB1 was a target of miR-449a in osteosarcoma. The overexpression of CCNB1 in osteosarcoma cells could suppress osteosarcoma cell proliferation induced by the miR-449a mimic. Their findings demonstrated that targeting miR-449a/CCNB1 could have significant clinical implications for treating osteosarcoma. It may represent a potential treatment strategy using engineered exosome-based therapies for the management of osteosarcoma.

With the rapid progress of the aging population in society, the prevalence of age-related diseases including osteoporosis has significantly increased and is now considered a serious public health issue (Hesari et al., 2023). Bone regeneration has attracted the attention of a wide range of researchers. The research conducted by Freitas et al. indicated that the expression of the lncRNA CASC2 is downregulated during the osteogenic differentiation of human bone mesenchymal stem cells (hMSCs). The results demonstrated that matrix mineralization of hMSCs was increased with lncRNA CASC2 downregulation. They further found that lncRNA CASC2 regulated the mineralization capacity of hMSCs through target protein COMP by proteomic analysis, and the inhibition of COMP could decrease mineral deposition in hMSCs. Their study clarified that regulating CASC2 levels is a potential approach for controlling bone mineralization.

In recent years, circular RNA has been found to play an important regulatory role in various diseases through multiple signal transduction pathways (Meng et al., 2023). The study conducted by Wu et al. showed that inhibition of hsa_circ_0008870 could significantly suppress chondrocyte proliferation and endochondral ossification. They further found that hsa_circ_0008870 regulates MAPK1 expression by sponge miR-185-3p in chondrocyte cells. The results indicated that the downregulated hsa_circ_0008870 decreased expression of MAPK1 by impairing the sponge action of miR-185-3p, thereby reducing chondrocyte proliferation and endochondral ossification, which results in a short stature phenotype. Their researcher revealed that hsa_circ_0008870 could be a potential therapeutic target for idiopathic short stature.

With the ongoing demographic changes, the number of elderly patients requiring free tissue transfer is increasing, for example, following cancer resection. Flap survival rates after transferring is always considered a vital clinical problem by the majority of researchers (Mirzabeigi et al., 2012; Chiu et al., 2017). Timely detection and intervention are crucial for saving free flaps, so close postoperative free flap monitoring is indispensable. Knoedler et al. reviewed currently available and employed methods for free flap monitoring, including clinical examination and handheld acoustic Doppler sonography. The review also summarized modern and innovative alternatives/adjuncts in free flap monitoring, such as color duplex ultrasonography, implantable Doppler, near-infrared spectroscopy, and hyperspectral imaging. The authors further analyzed free flap monitoring methods, including thermal imaging, oxygen partial pressure, and skin paddles, and their potential in elderly patients.

In summary, age-related diseases have consistently been one of the global focal points in health research. This Research Topic contains innovative therapeutic strategies for age-related diseases. Recently, due to good stability and low immunogenicity, stem cell-derived exosomes served as a promising candidate for tissue regeneration (Hu et al., 2021a; Hu et al., 2021b). In the future, there will be more new approaches and strategies to treat age-related diseases.
